# Cell Wall Assembly and Intracellular Trafficking in Plant Cells Are Directly Affected by Changes in the Magnitude of Gravitational Acceleration

**DOI:** 10.1371/journal.pone.0058246

**Published:** 2013-03-13

**Authors:** Youssef Chebli, Lauranne Pujol, Anahid Shojaeifard, Iman Brouwer, Jack J. W. A. van Loon, Anja Geitmann

**Affiliations:** 1 Institut de recherche en biologie végétale, Département de sciences biologiques, Université de Montréal, Montréal, Québec, Canada; 2 Epona Medical, Rotterdam, The Netherlands; 3 Department of Craniofacial Surgery & Oral Cell Biology, Academisch Centrum Tandheelkunde Amsterdam (ACTA), University of Amsterdam and Vrije Universiteit Amsterdam, Research Institute MOVE, Amsterdam, The Netherlands; 4 Life and Physical Sciences Instrumentation and Life Support Section (TEC-MMG), European Space Agency (ESA), Noordwijk, The Netherlands; Wuhan University, China

## Abstract

Plants are able to sense the magnitude and direction of gravity. This capacity is thought to reside in selected cell types within the plant body that are equipped with specialized organelles called statoliths. However, most plant cells do not possess statoliths, yet they respond to changes in gravitational acceleration. To understand the effect of gravity on the metabolism and cellular functioning of non-specialized plant cells, we investigated a rapidly growing plant cell devoid of known statoliths and without gravitropic behavior, the pollen tube. The effects of hyper-gravity and omnidirectional exposure to gravity on intracellular trafficking and on cell wall assembly were assessed in *Camellia* pollen tubes, a model system with highly reproducible growth behavior *in vitro*. Using an epi-fluorescence microscope mounted on the Large Diameter Centrifuge at the European Space Agency, we were able to demonstrate that vesicular trafficking is reduced under hyper-gravity conditions. Immuno-cytochemistry confirmed that both in hyper and omnidirectional gravity conditions, the characteristic spatial profiles of cellulose and callose distribution in the pollen tube wall were altered, in accordance with a dose-dependent effect on pollen tube diameter. Our findings suggest that in response to gravity induced stress, the pollen tube responds by modifying cell wall assembly to compensate for the altered mechanical load. The effect was reversible within few minutes demonstrating that the pollen tube is able to quickly adapt to changing stress conditions.

## Introduction

The evolution of plants has been driven by different biotic and abiotic stresses that have varied in time and space. Gravity is the only constant factor to which plants had to adapt in a permanent manner. The first plants to appear on Earth were mostly composed of soft tissues, devoid of any hard vascular tissue and plant life was therefore limited to buoyant environments. The colonization of terrestrial environments was accompanied by the development of robust vascular systems and stiff cell walls, which enabled plants to build aerial organs rising in the direction opposite to the graviational force. Since the appearance of vascular terrestrial plants around 440 million years ago, plants have evolved by adapting permanently to this constantly present force [1]. To withstand the mechanical load imposed by gravity, plants developed two strategies, one being based on the erectile force created by the balance between the internal turgor pressure and the tensile resistance of the primary cell wall, the second consisting in the production of stiff secondary walls [2]. For both mechanisms, the cell wall, its timely assembly and its mechanical properties are essential regulatory factors. The plant cell wall (or extracellular matrix) is made of interlinked networks of polysaccharide polymers with distinctive and modifiable mechanical properties. The relative abundance and the types of polymers as well as the interpolymer linkages are controlled to ensure functional growth of plant organs and differentiation of the individual cell. Therefore, the polymers composing the cell wall have to be delivered to or synthesized directly at the cell surface under tight spatial and temporal control [3]. Any environmental change has the potential to disrupt this process, resulting in abnormal morphological phenotypes and/or dysfunctional growth and differentiation. Changes in the magnitude of the gravitational acceleration have been shown to disrupt growth of organs and cells, for example during *Arabidopsis* hypocotyl growth [4], root elongation [5] and seed formation [6]. Many of these phenotypes have been related to defects in cell wall assembly.

In order to respond to gravity-related stress, the cell or organism must actually be able to perceive it. Two major models have been proposed to explain how plant cells perceive gravity -induced stimulation, the statolith model and the gravitational pressure model (reviewed in [1], [7]). Statolith-based mechanisms have been studied in great detail [1], but most plant cells are not equipped with these specialized organelles. Cells devoid of statoliths do nevertheless respond to a gravi-stimulus, however, and hence the gravitational pressure model or alternative concepts that explain gravi-perception in the absence of statoliths warrant further research to understand how plants are affected by and respond to gravity or the absence thereof [7].

In order to understand how non-statocyte plant cells are affected by gravity, we analyzed the effect of altered gravitational acceleration on intracellular transport and cell wall assembly in a non-statocyte and non-gravitropic plant cell system. In most plant cell systems, cell wall deposition occurs over days or even weeks and effects of changes in the magnitude of gravitational acceleration are only visible after long term exposure to the stress. We therefore opted to study the fastest growing cell in the plant kingdom, the pollen tube. The pollen tube is a cellular protrusion formed by the pollen grain upon contact with a receptive stigma, the landing platform on top of the pistil. The pollen tube is responsible for fertilization in higher plants and hence crucial for seed and fruit formation. Pollen tubes, similar to root hairs, fungal hyphae and neurons, are tip-growing cells. They are characterized by a uni-directional growth pattern, which significantly facilitates quantification of the growth process and geometrical parameterization compared to cells expanding in two or three dimensions. The growth rates of pollen tubes can be up to hundreds of micrometers per minute. Sustaining this process requires continuous synthesis of cell wall precursors and their delivery to the cell surface by exocytosis. Unlike most other plant cells, tip growing cells focus their exocytosis activity on a very small area on the cellular surface, the expanding apex. To ensure morphogenesis of a perfectly cylindrical shape, vesicle exocytosis occurs at high rates and is spatially and temporally controlled by a multitude of parameters [8], [9]. Importantly, the pollen tube does not have any known statoliths, nor does it display a gravitropic response [10] which is in accordance with its physiological behavior as the flowers of many plant species are oriented arbitrarily relative to the gravity vector. To find their target, the female gametophyte, the elongating pollen tube has to follow chemical and mechanical cues presented by the pistillar tissues and the female gametophyte. A gravitropic response would interfere with the chemotropic and thigmotropic behavior necessary for target finding.

The rapid growth rate of the pollen tube allows for the observation of rapid and easily visible cellular responses upon mechanical or chemical manipulation [8], [9]. This rapid response time of the pollen tube also applies to gravity stress [11], [12] and allows fitting experiments into short time frames unlike experimentation on plant cell types with slower metabolism.

Here, we studied the effects of changes in the magnitude of gravitational acceleration on the behaviour of pollen tube growth, cell wall assembly and vesicle trafficking. We grew *Camellia japonica* pollen tubes exposed to omnidirectional gravity (omnidirectional-*g*; also known as simulated microgravity) and hyper-gravity (hyper-*g*) conditions at the facilities of the European Space Research and Technology Centre (ESTEC) of the European Space Agency (ESA) located in The Netherlands. Using immuno-cytochemical labelling coupled with epi-fluorescence microscopic techniques, we quantitatively analyzed the biochemical composition and spatial distribution of the different cell wall components in pollen tubes grown at different magnitudes and directions of effective gravitational acceleration ranging from omnidirectional-*g* to hyper-*g*. To further understand the effect of altered gravitational acceleration on cell wall assembly and morphogenesis, we assessed how intracellular vesicle trafficking is modulated by changes in *g*-acceleration. This approach required live cell fluorescence imaging, which was performed with an inverted microscope equipped for epi-fluorescence imaging installed as payload on the Large Diameter Centrifuge (LDC) at ESTEC-ESA.

## Materials and Methods

### Plant Material

Pollen was collected directly after anthesis from a *Camellia japonica* plant grown in the greenhouse of the Montreal Botanical Garden. To minimize artefacts due to varying maturity of pollen grains, only batches collected during the same week were used for any given series of experiments. Pollen grains were then dehydrated over silica gel for 24 hours and stored at −20°C until use.

### Pollen Culture

Pollen grains were hydrated for 30 min and suspended in a growth medium containing 100 µg ml^−1^ H_3_BO_3_, 300 µg ml^−1^ Ca(NO_3_)_2_, 100 µg ml^−1^ KNO_3_, 200 µg ml^−1^ MgSO_4,_ and 50 mg ml^−1^ sucrose. Unless stated otherwise, the pollen grain suspension was deposited in the wells of 0.4 µm Ibidi® plates (µ-Slide VI 0.4, IbiTreat) and incubated under omnidirectional-*g*, 1 *g*, and hyper-*g* up to 20 *g*.

### Viability Test

Pollen grain viability was assessed using fluorescein diacetate (FDA) from a stock solution dissolved in acetone at 10 mg mL^−1^ and stored at −20°C. FDA was diluted in a 5% sucrose solution to a final concentration of 0.2 mg mL^−1^. Hydrated pollen was suspended in 250 µL of the FDA solution on a glass slide and kept in the dark for 5 min. Observations were made with a Zeiss Imager-Z1 microscope with excitation light at 470 nm and a 515–565 nm band pass emission filter. Under these conditions, only viable pollen grains emit a fluorescence signal.

### Omnidirectional Exposure to Gravity

During omnidirectional exposure to gravity, the orientation of the specimen relative to the vector of the Earth’s gravity force is arbitrarily changed over time. To achieve this, pollen grains were germinated in Ibidi® cells fixed to the stage of a Random Positioning Machine (RPM) [13] at the European Space Research and Technology Centre (ESTEC) of the European Space Agency (ESA). The RPM consists of two independently rotating frames and was operated in random modes of speed, direction and interval. The maximum acceleration was set at 60° s^−1^ via a computer user interface with dedicated control software. The samples were fixed in the centre of the inner frame with a resulting largest radius of 70 mm to the outermost wells resulting in a maximum residual *g* due to rotation of less than 10^−4^ *g* [13]. After 3 hours, pollen tube growth was stopped by adding a 37% formaldehyde solution (Sigma-Aldrich) and subsequently labelled as mentioned below.

### Hyper-gravity Conditions for Pollen Tube Cell Wall Labelling

Hyper-*g* was simulated by centrifugation using a centrifuge built in-house. Briefly, Ibidi® cells were fixed on a circular plate attached to a rotor turning at an adjustable, computer-controlled speed. Values of effective gravitational acceleration experienced by the cells were altered by modifying *RPM* (revolutions per minute) of the plate and taking into account the distance *r* from the center of the plate to the sample using. 
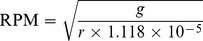



The term gravitational acceleration used in this paper denotes the resultant of the acceleration induced by this rotation and that caused by the Earth’s gravitational field. Pollen tubes were grown for 2 to 3 hours under hyper-*g* conditions and subsequently fixed and labelled as detailed below.

### Fixation and Fluorescence Label

All steps were carried out in a microwave oven (Pelco BioWave® 34700 equipped with a Pelco Cold Spot®) operating at 150 W under 53.34 cm of Hg vacuum at a controlled temperature of 30±1°C. Pollen tubes where fixed after 2 hours of germination under hyper-*g* conditions. Fixative was added directly to the growing tubes immediately after the centrifugation or after a recovery period on the bench of 5, 10 and 15 min. Fixation was performed in 3.5% w/v freshly prepared formaldehyde and 0.3% v/v glutaraldehyde in Pipes buffer (50 mM Pipes, 1 mM EGTA 0.5 mM, MgCl_2_, pH = 6.9) for 40 seconds followed by 3 washes in Pipes buffer. For immuno-labelling pollen tubes were then washed 3 times with phosphate buffer saline (135 mM NaCl, 6.5 mM Na_2_HPO_4_, 2.7 mM KCl, 1.5 mM KH_2_PO_4_, pH 7.3) containing 5% w/v bovine serum albumine (PBS-BSA). All subsequent washes were done with PBS-BSA for 40 seconds. All antibodies were diluted in PBS-BSA and incubations were done for 7 min followed by three washes in buffer. Controls were performed by omitting incubation with the primary or the secondary antibody. Pectins with low and high degree of esterification were labelled with monoclonal antibodies JIM5 and JIM7, respectively (diluted 1∶50; Paul Knox, University of Leeds, United Kingdom), followed by Alexa Fluor 594 anti-rat IgG (diluted 1∶100; Molecular Probes). Callose was labelled with a monoclonal IgG antibody to (1→3)-β-glucan (diluted 1∶200; Biosupplies Australia Pty Ltd) followed by Alexa Fluor 594 anti-mouse IgG (diluted 1∶100; Molecular Probes). Label for crystalline cellulose was performed with Cellulose Binding Module 3a (diluted 1∶200; Paul Knox, University of Leeds, United Kingdom) followed by a monoclonal mouse-anti-polyhistidine antibody (diluted 1∶12; Sigma) and subsequent incubation with Alexa Fluor 594 anti-mouse IgG (diluted 1∶100; Molecular Probes).

### Fluorescence Microscopy

Differential interference contrast (DIC) and fluorescence micrographs were acquired with a Zeiss LSM 510 META/LSM 5 LIVE/Axiovert 200 M system. Localization of Alexa 594 was done with a 561 nm laser and an emission filter LP 575. To ensure image acquisition in the linear range, exposure times were adjusted for each image so that only one or two pixels were saturated. Z-stacks of 1 µm interval were acquired for image reconstruction using the Zeiss LSM 5 Duo software.

### Image Processing and Fluorescence Quantification

ImageJ software (Rasband, W.S., ImageJ, U. S. National Institutes of Health, Bethesda, Maryland, USA, http://rsb.info.nih.gov/ij/, 1997–2012) was used for quantification of fluorescence intensity based on maximum intensity projections of Z-stacks. Pixel intensity was measured along the periphery of each pollen tube, beginning from the pole (the outermost tip of the tube). Values for fluorescence intensity were normalized to the highest value present on an individual tube before averaging over all tubes (n>10 for each sample). Values on the x-axis in the graphs represent the meridional distance from the pole of the cell. Given a typical tube diameter of 14 µm and an approximately hemisphere-shaped apex for *Camellia*, a distance of 14 µm on the meridional curvature corresponds to a tube length of 10 µm measured along the longitudinal axis. For each treatment, at least 10 tubes were analysed. Each experiment was repeated at least 3 times.

### Live Imaging of Vesicle Trafficking in Pollen Tubes Grown in Hyper-gravity

**Figure 1:**
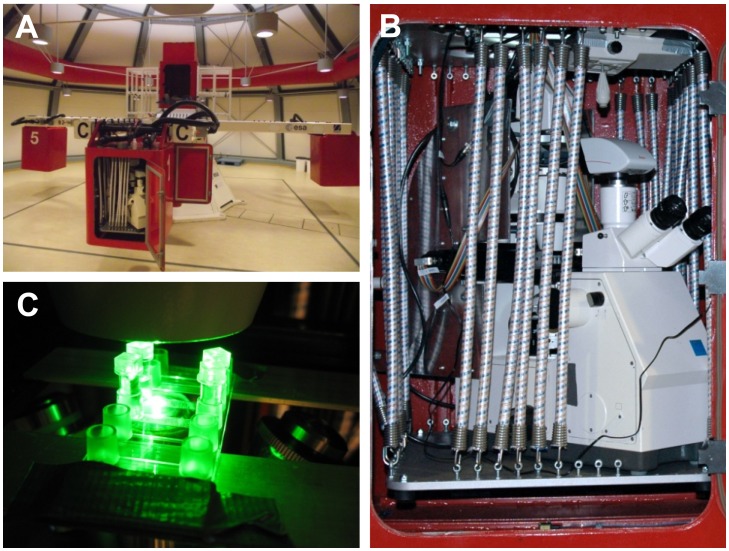
Large Diameter Centrifuge and experimental setup. (A) The LDC is located at the research facilities of the European Space Research and Technology Centre of the European Space Agency in Noordwijk, The Netherlands. It is composed of four arms supporting a total of up to 6 gondolas. (B) An inverted Zeiss Axiovert 100 microscope equipped with a mercury lamp was fixed inside one of the gondolas allowing live observations of growing pollen tubes in Ibidi® cells (C).

To allow for live cell imaging at hyper-*g*, the Large Diameter Centrifuge (LDC) housed at the ESTEC-ESA [14] was used. The LDC has four arms supporting up to 6 gondolas with a maximum payload of 80 kg each. The total diameter of the device is 8 m and *g*-acceleration of up to 20 *g* can be achieved. An inverted Zeiss Axiovert-100 microscope equipped for epi-fluorescence imaging was fixed in one of the gondolas using elastic bungee cords (Fig. 1). The x-y position of the stage and the focus of the microscope were remote controlled. The microscope was equipped with a Leica DFC 300 FX digital camera for brightfield and fluorescence imaging.

For the visualization of vesicle trafficking, the styryl dye FM1–43 was added at a final concentration of 1 µg mL^−1^ to growing *Camellia* pollen tubes. For FM1–43 imaging, a filter set with excitation BP 450–490 nm, beam splitter FT 510 nm and emission LP 515 nm was used. Imaging was performed on growing pollen tubes at 1, 2, 5, 7, 10 and 12 *g*. Images were acquired at 1 frame s^−1^.

## Results

### A Change in Gravitational Acceleration Affects Pollen Germination, Pollen Tube Diameter, Growth Rate and Surface Expansion

In previous studies, *Arabidopsis thaliana* and *Brassica rapa* pollen tubes grown under hyper-*g* conditions were found to display drastically reduced growth rates and aberrant tube morphologies [15]. To assess the general effect of gravitational acceleration and magnitude on *Camellia* pollen performance we quantified the percentage of pollen germination, pollen tube growth rate, pollen tube diameter and pollen tube surface expansion rate under various hyper-*g* and under omnidirectional-*g* conditions.

**Figure 2:**
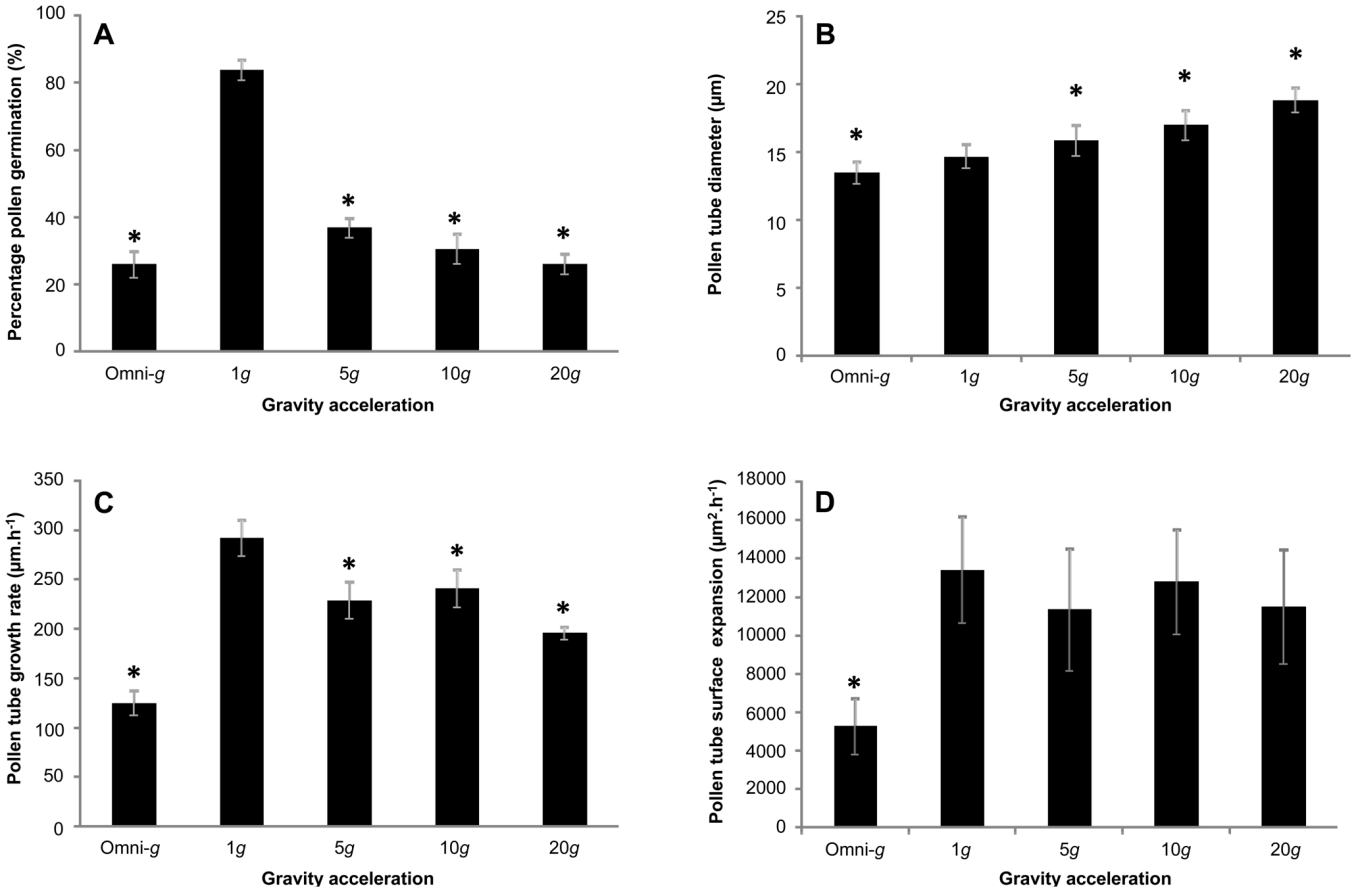
Response of *Camellia* pollen tube morphology to altered gravitational acceleration. (A) Germination percentage of pollen grains. Pollen grains were considered germinated when the length of the pollen tube exceeded the diameter of the pollen grain. (B) Pollen tube diameter was measured at approximately 20 µm from the pole. (C) Pollen tube elongation rate as calculated from pollen tube length after 2 to 3 hours following imbibition. (D) Rate of surface expansion as calculated from pollen tube diameter and elongation rate. Asterisks (*) indicate statistically significant difference between samples grown in altered gravity conditions compared to the samples grown at 1 *g* (Two way t-test yielded p<0.001).

In the control experiments at 1 *g*, an average of 80% of the *Camellia* pollen grains were able to germinate. This value was close to the viability of the grains tested using FDA of 90% ±3.4%, demonstrating that our *in vitro* growth conditions are close to optimal. The effect of both increased gravitational acceleration and exposure to omnidirectional-*g* was dramatic as germination percentage decreased by more than half in both situations (Fig. 2A).

In our *in vitro* growth conditions, *Camellia* pollen tubes germinated at 30 min after contact with the growth medium and elongated at an average rate of 290 µm h^−1^ as calculated from pollen tube length of germinated pollen grains at 3 hours after imbibition. *Camellia* pollen tubes growing at 1 *g* had an average diameter of 14±0.92 µm. When tubes were grown under omnidirectional-*g* conditions, the diameter of the pollen tubes was reduced by 8% and under hyper-*g* conditions the diameter of the pollen tubes increased by 8% at 5 *g* and 38% at 20 *g* (Fig. 2B) as measured in the cylindrical shank of the tube.

Quantification of pollen tube length at hyper-*g* showed that those pollen tubes that were able to germinate under these conditions, were somewhat affected in their ability to elongate, but not dramatically so. Only at a gravitational acceleration value as high as 20 *g* was the growth rate reduced to half of that of the bench control. Upon omnidirectional exposure to gravity, reduction of the growth rate was significant to less than half of the control value (Fig. 2C).

Since both growth rate and diameter were altered by changed gravitational acceleration, we determined the overall expansion rate of the cell surface (consisting of plasma membrane and cell wall). At 1 *g* the surface of *Camellia* pollen tubes increased by 13 377±2 783 µm^2^ h^−1^. While under hyper-*g* this surface expansion rate did not change significantly, it was reduced by 39% under omnidirectional-*g* (Fig. 2D).

### A Change in Gravitational Acceleration does not Affect the Spatial Profile of Pectin Distribution

**Figure 3:**
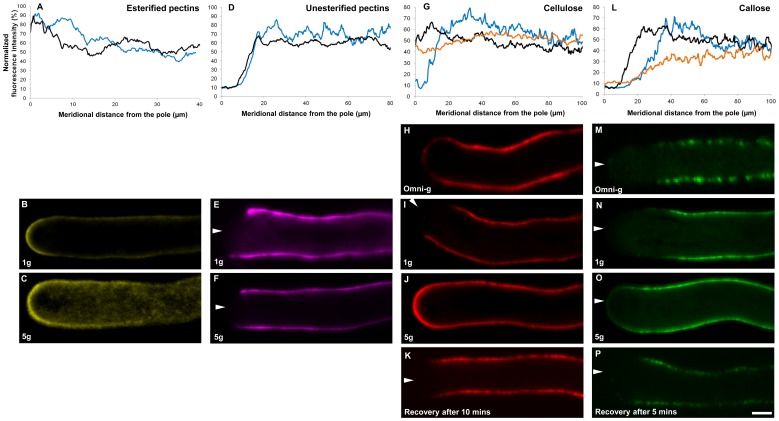
Effect of gravity stress on spatial distribution profile of cell wall components. Spatial distribution of esterified pectins (A,B,C), unesterified pectins (D,E,F), cellulose (G,H,I,J,K), and callose (L,M,N,O,P) in *Camellia* pollen tubes grown under omnidirectional-*g* (orange curve), 1 *g* (blue curve) and 5 *g* conditions (black curve). Relative label intensities were quantified along the meridional tube surface measured on z-stack projections and normalized to the highest pixel intensity before averaging (n>10 tubes per sample) (A,D,G,L). Corresponding typical fluorescence micrographs of median optical sections (B,C,E,F,H,I,J,M,N,O) are displayed for the different *g*-accelerations. Recovery of the typical spatial profile observed at 1 *g* for cellulose and callose was observed at 5 min after removal from the centrifuge (K,P). Specific label was performed using JIM7 and JIM5 antibodies, directed against highly esterified and unesterified pectins, respectively, Cellulose Binding Module 3A against crystalline cellulose and anti (1→3)-β-glucan against callose. Arrowheads mark the position of the pollen tube tip where invisible. Bar = 10 µm.

Pollen tubes are characterized by a precisely controlled, spatially defined distribution of cell wall components [16], [17]. Pectins form a continuous layer around the tube, but they are highly methyl-esterified at the apex and acidic in the tubular shank [9]. This chemical gradient is the result of enzymatic deesterification upon the maturation of the cell wall [18]. Deesterification is thought to allow gelation of pectin polymers by calcium ions, a process that stiffens the wall [19], [20], [21]. In addition to pectin gelation, the tubular shank of the tube is also rigidified by the deposition of callose [22] and cellulose [23]. This maturation of the cell wall in longitudinal direction prevents the distal cell wall from expanding [16], [24]. In particular, the cross-over point between methyl-esterified and non-esterified pectins has been proposed to define the transition region between the hemisphere shaped tip and the cylindrical shank of the tube [16], [25]. Therefore, the observed change in tube diameter upon modulation of the effective gravitational acceleration motivated us to assess whether the spatial distribution of the cell wall components was affected in these tubes. We hypothesized that in larger tubes formed under hyper-*g* conditions the cross-over point between the two configurations of pectin would be further away from the pole, whereas in smaller tubes resulting from omnidirectional-*g* conditions this cross-over point was expected to be closer to the tip. However, immunohistochemical label with JIM5 and JIM7, specific antibodies against pectins with low and high degree of esterification, respectively, revealed that the characteristic spatial profile of pectin was not visibly affected by the gravitational acceleration values and directions tested here (Figs. 3A, B, C, D, E, F). Quantification of relative fluorescence levels along the tube periphery did not yield any detectable difference in the spatial distribution of either type of pectin between pollen tubes grown at 1 *g* and pollen tubes exposed to hyper-*g*.

### A Change in Gravity Conditions Causes the Relocalization of Cellulose Towards the Apex of the Tube

Since the spatial profile of pectins was not altered as a result of increased gravity-stress, we hypothesized that instead the other cell wall components might be affected in their distribution. Although crystalline cellulose is not generally present in high quantities in the pollen tube cell wall [26], [27], it was found to play a crucial role in determining the pollen tube shape of *Petunia hybrida, Lilium auratum, Lilium orientalis, Solanum chacoense* and *Picea abies* [23], [28], [29]. To detect crystalline cellulose it was labelled with cellulose binding module 3a (CBM3a) [30]. After two hours of growth at 1 *g*, crystalline cellulose was only detected in the distal region of *Camellia* pollen tubes starting at 10 µm from the pollen tube tip. Along the tube, the abundance of cellulose increased steeply with half-maximum fluorescence intensity at 12 µm and reached a plateau at 20 µm (Figs. 3G,I). When pollen tubes were subjected to omnidirectional-*g* or hyper-*g* conditions of 5 *g* and higher, crystalline cellulose deposition changed dramatically. In both situations the fluorescence label was uniform around the entire cell perimeter, including the tip (Figs. 3G,H,J). No noticeable differences in this ectopic cellulose localization were observed between various hyper-*g* levels (Figure S1A).

### Callose is Closer to the Tip in Tubes Grown in Hyper-gravity

Callose is the most abundant polymer in the pollen tube cell wall [26] and plays an important mechanical role in reinforcing it against compression and tension stresses in the distal part of the tube [22]. In *Camellia* pollen tubes grown at 1 *g*, visible deposition of callose appeared at 15 µm from the pole with the half-maximum fluorescence intensity at 30 µm meridional distance from the tip and a steep increase to a plateau at 40 µm (Figs. 3L,N). When pollen tubes were exposed to omnidirectional-*g*, callose deposition along the longitudinal axis increased more gradually with a half-maximum at 30 µm and a very gradual increase to a plateau at approximately 80 µm (Figs. 3L,M). Remarkably, the callose layer was not smooth as in the 1 *g* control, but irregular with a speckled appearance (Figure S2). By contrast, in the 5 *g* sample, callose deposition appeared as a smooth layer starting closer to the tip, beginning at 7 µm with a half-maximum at 17 µm and a plateau at 21 µm (Figs. 3L,O). No noticeable difference in callose localization was observed between different hyper-*g* levels (Figure S1B).

To further confirm that the mis-localization of cellulose and callose was due to a change in hyper-*g* conditions and that the effect was reversible, pollen tubes were grown at 5 *g* for 2 hours, removed from the centrifuge and then left to grow at 1 *g* for 5, 10 or 15 min before fixation and immunolabel. As few as 5 min at 1 *g* were sufficient for cellulose and callose profiles to return to the characteristic distances from the apex that were observed in samples grown in parallel at 1 *g* for the entire time (Figs. 3K,P).

### Vesicle Trafficking is Reduced at Hyper-gravity

**Figure 4:**
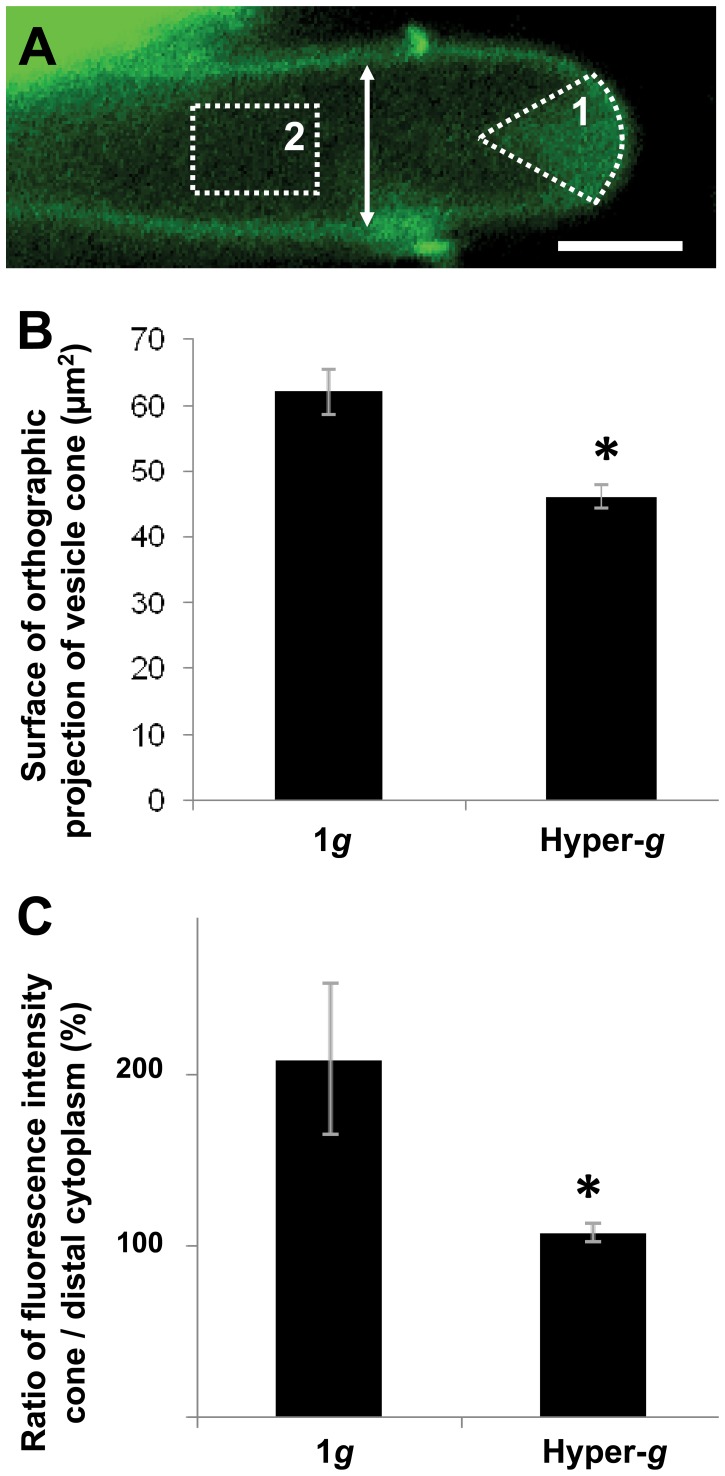
Intracellular trafficking is reduced at hyper-gravity. Geometry (A,B) and fluorescence intensity (A,C) of the apical vesicle cone. Vesicles were labelled with the styryl dye FM1–43 taken up by endocytosis. At the time of imaging the dye had been incorporated into most of the endomembrane system including exocytotic vesicles. (A) represents a pollen tube marked with FM1–43, the orange arrow represents the diameter of the pollen tube. Surface area of orthographic projection of the vesicle cone (B) and fluorescence intensity (area 1 in A) as well as the fluorescence intensity of the distal cytoplasm (area 2 in A) were measured in pollen tubes grown at 1 *g* and in hyper-*g* conditions. Relative fluorescence intensity (C) was expressed as a ratio between the fluorescence of the vesicle cone over the fluorescence of the distal cytoplasm fluorescence of the same tube. Asterisks (*) indicate statistically significant difference between samples grown in hyper-*g* and control samples grown at 1 *g* (Two way t-test yielded p<5.10^−4^ for the three graphs). Bar = 10 µm.

The principal process responsible for pollen tube expansion is the continuous and extremely high exocytosis rate of vesicles containing cell wall precursors and enzymes responsible for cell wall synthesis. Exocytosis is accompanied by membrane endocytosis to ensure a balanced deposition rate of extracellular matrix and membrane material. Pollen tube growth is thus characterized by a tight temporal and spatial control of the dynamic vesicular trafficking [31]. Since the spatial profiles of cellulose and callose were affected by altered gravitational acceleration, and since these cell wall components are assembled by synthases delivered to the plasma membrane by exocytosis, we wanted to understand how hyper-*g* affects intracellular trafficking. To this end, pollen tubes were grown in the presence of the styryl dye FM1–43 and exposed to increased gravitational acceleration in a Large Diameter Centrifuge allowing for live cell imaging in brightfield and fluorescence mode. In *Camellia* pollen tubes grown at 1 *g*, vesicles tended to aggregate in the apical cytoplasm in a volume with the shape of an inverted cone. This phenomenon has been described in many other angiosperm species [31], [32], [33] and is generated by a reverse fountain shaped transport pattern mediated by the actin cytoskeleton [34], [35]. Previously, we had quantified this streaming pattern using spatio-temporal image correlation spectroscopy (STICS) to circumvent the fact that the sub-diffraction size of the vesicles precludes their tracking when densely packed and moving rapidly [31]. However, STICS requires high temporal resolution image series acquired by confocal laser scanning microscopy. The present setup in the LDC only allowed for conventional epi-fluorescence imaging making the quantification of vesicle movement with STICS impossible. To quantitatively assess the intensity of vesicle trafficking, we therefore determined the size of the inverted vesicle cone by determining the surface area of its orthographic projection in side view (i.e. the surface it occupies on an epi-fluorescence micrograph) as well as its fluorescence intensity. At 1 *g* he projection of the inverted vesicle cone occupied an average surface area of 62±10.0 µm^2^. The width of the cone measured as arc length at the tip was 9.7±1.9 µm. Under hyper-*g* conditions, the surface of the cone was reduced by 25.6% with small, non significant variations between the different hyper-*g* levels tested (between 5 *g* and 10 *g*) (Fig. 4A,B). The width of the cone did not vary significantly between different hyper-*g* levels or compared to the 1 *g* control.

To quantitatively determine the relative density of vesicles within the cone while excluding potential artifacts due to bleaching, we calculated the ratio between fluorescence intensities in the cone and in the distal cytoplasm of individual pollen tubes (Figs. 4A,C). Tubes growing at 1 *g* showed a cone/cytoplasm ratio of 2.1 whereas their counterparts grown at hyper-*g* showed a ratio close to 1.1 (with insignificant variations between the different values for gravitational acceleration values tested here), corresponding to a decrease of 48.5% of the cone/cytoplasm fluorescence ratio of the tubes grown in hyper-*g* compared to the tubes grown at 1 *g* (Fig. 4C).

## Discussion

### Altered Gravitational Acceleration Affects Pollen Performance

Our data showed that in *Camellia* pollen tubes grown under altered gravitational acceleration both germination percentage and growth rate were reduced. These findings are in accordance with observations made in pollen grains from *Arabidopsis* [15], *Pyrus* and *Prunus* [10] germinated under hyper-*g* conditions and suggest that despite the absence of an obvious gravity perception mechanism, certain cellular functions are affected when these cells are exposed to altered gravitational acceleration. Interestingly, the germination percentage was reduced more dramatically than pollen tube growth, since those tubes that succeeded to germinate did grow relatively well, even at gravitational acceleration values up to 20 *g*. These data indicate that the mechanism of germination might require cellular processes that are more susceptible to mechanical stress than those governing pollen tube growth. Whether this sensitivity of the germination process to gravity stress is related to the critical step of the initial polarization of the cell [36] or to the processes that lead to the softening of the aperture cell wall required for pollen tube outgrowth [37] remains to be investigated.

### Intracellular Trafficking is Altered at Hyper-gravity

The pollen tube is a tip growing cell, that elongates through a constant supply of cell wall material to the growing apex of the tube. The delivery of this material relies on an exquisitely regulated balance between exocytosis and endocytosis both of which need to be precisely controlled in time and space [31]. Intracellular trafficking is therefore of crucial importance for proper pollen tube elongation and the process is governed by a characteristic actin array [35] that directs vesicles to the expanding apical cell surface [33]. Using fluorescence intensity of vesicles labelled by the styryl-dye FM1–43 as a proxy for trafficking, we observed a significant reduction in the number of vesicles present in the apical cone at hyper-*g* compared to the 1 *g* control. This result is consistent with findings by Lisboa and colleagues [12] who used samples fixed after exposure to temporary hyper-*g* stress and showed that endocytotic retrieval of plasma membrane in tobacco pollen tubes was reduced compared to the 1 *g* control. At the hyper-*g* values tested here, there was no significant dose-dependent effect, however. Whether the reduction of vesicles in the apex in our setup is due to a slower rate of overall intracellular transport or a reduced number of secretory vesicles produced cannot be deduced from the present data. A reduced number of vesicles in the apical cone could also mean that the equilibrium between the forward and rearward transport rates within the tube is altered. If the rearward vesicle transport by the central actin array is accelerated, or the forward transport in the periphery is slowed, the accumulation of vesicles in the apex would be reduced [35]. The fact that the surface expansion rate of pollen tubes grown at hyper-*g* was comparable to their counterparts grown at 1 *g* shows that despite the difference in the rate of longitudinal elongation, the same amount of cell wall material is deposited at the apex over time (assuming that cell wall thickness is constant). This result suggests that the number of exocytosis events is not affected by the hyper-*g* stress, but rather that the location of these events, and thus the resulting morphogenetic processes are altered. The wider tube diameter at hyper-*g* is indicative either of a less focused distribution of exocytosis events or of the subcellular localization of endocytosis being shifted further distal. Remarkably, the unaltered rate of surface growth suggests that the vesicle fusion rate is indeed a rate limiting factor for pollen tube expansion - independently of the degree of polarity and precise morphology of the resulting cell.

### Gravity Stress Affects Cell Wall Assembly and Morphogenesis

As a consequence of gravity stress, the diameter of *Camellia* pollen tubes was altered and this effect was clearly dose-dependent. Exposure to omnidirectional-*g* resulted in a reduced diameter, whereas increasing the magnitude of gravitational acceleration lead to an increasingly large pollen tube diameter. The diameter of the pollen tube is determined by the kinetics of rigidification of the cell wall material once it is deposited at the apex [16], [24], [38]. The stiffening is caused by calcium gelation of de-methyl-esterified pectins. Pectin de-methyl esterification is accomplished by pectin methyl-esterase (PME) that is equally secreted at the apex, but whose activity is inhibited in the apical dome by the PME inhibitor (PMEI) [25]. It is thought that endocytosis of the inhibitor at the transition region between the apex and the shank allows the enzyme to act and hence the spatial location of this endocytotic activity might be a critical determinant for pollen tube diameter and shape generation. A disturbance either in the spatial or temporal control of the secretion of the amount of pectin, the amount of PME or of the efficiency or location of endocytosis of the PMEI could provide possible explanations for a change in cellular diameter.

If an altered kinetics in pectin de-esterification were the principal reason for the observed altered tube diameter, one would predict that the pectin profiles (both esterified and non-esterified) should be shifted slightly - towards the apex to explain the smaller pollen tube diameter at omnidirectional-*g* and away from the apex at hyper-*g*. The latter shift would be expected to be as small as 3.1 µm at 20 *g* in accordance with the observed 4 µm increase in cellular diameter compared to the 1 *g* control. However, the high variability in the pectin distribution profiles between individual pollen tubes, and the fact that these profiles only represent relative distributions, rather than absolute pectin abundance, did not allow us to discern such a small shift.

### Hyper-gravity Affects Cellulose Assembly

In normally growing *Camellia* pollen tubes, both cellulose and callose are absent from the apical region of the cell. This characteristic profile was changed dramatically as cellulose appeared in the tip both at hyper-*g* and omnidirectional-*g*. In general, cellulose is known to stiffen the plant cell wall and its absence from the pollen tube tip was thought to be consistent with the rapid expansion activity occurring in this cellular region. The fact that the gravity stressed tubes displaying apical cellulose only showed moderately reduced growth rates raises questions about the concept of wall stiffening through cellulose. As long as the cellulose microfibrils are short or not cross-linked, their presence does not necessarily imply a stiffening of the wall. This explanation would be consistent with the fact that not all pollen tubes growing under normal *in vitro* conditions are devoid of cellulose at the apex. Those of *Arabidopsis thaliana* and *Lilium orientalis*, for example, display crystalline cellulose at the apex [16], [17] suggesting that the role of this polymer for the morphogenetic process may be more subtle. It remains to be elucidated how the gravity-trigger causes cellulose to appear at the apex in *Camellia* pollen tubes. The explanation may be simply in the kinetics of the cellulose synthase activity that could be argued to be time-dependent rather than position-dependent. Since the tubes grow slower under gravity-induced stress, the amount of cellulose formed at a given distance from the pole of the cell may be higher as a result of this location being distanced from the advancing pole at a reduced rate. In this scenario, the altered cellulose profile is a passive consequence of a lower growth rate. Alternatively, one can hypothesize that additional deposition of cellulose at the apex serves to stabilize an otherwise weakened cell wall. This weakening might for example be induced by a reduced supply with pectin, consistent with the reduced vesicle trafficking. It is known that pollen tubes are able to compensate for the lack of one cell wall component by increased deposition of another [23]. This scenario would therefore represent an active response of the cell to a modulation in cell wall mechanics. As a third hypothesis one could argue that rather than affecting cell wall assembly directly, because of a cellular flattening induced by the centrifugal force, the hyper-*g* induced stress simulates an increase in turgor leading to an increase in tensile stress in the wall. An effort to strengthen the apical wall could therefore be interpreted to serve to counteract the increased turgor and prevent bursting. In this latter scenario, it is difficult to explain why cellulose would equally appear at the tip at omnidirectional-*g*, which is generally interpreted to correspond to simulated microgravity. The finding could be reconciled with this hypothesis, on the other hand, if one recognizes that omnidirectional-*g* is, in fact, not the removal of gravity, but simply a continuous change of the orientation of the 1 *g* gravitational acceleration that might result in increased overall stress than the static positioning in the control sample.

The rapid reversibility of the altered spatial profiles of cellulose and callose after removal of the hyper-*g* induced stress confirms that the phenomenon is caused by a direct effect of gravity stress on the cellular processes involved in cell wall delivery and assembly rather than an effect mediated by a change of gene expression or synthesis of cell wall material. Full recovery was achieved within 5 min, a time period that is unlikely to involve any processes that are significantly upstream of the growth machinery, such as signaling to the nucleus or transcriptional regulation.

### Omnidirectional Application of Gravity Disrupts Callose Deposition

In normally growing pollen tubes, callose is deposited in the distal portion of the tube and plays a role of reinforcement against compression and tension stresses [22]. At hyper-*g*, although the apex was still devoid of this polymer, the callose layer was visible closer to the tip than at the 1 *g* control. As was the case for cellulose, a kinetics-based argument could be brought forward for this phenomenon. At lower growth rate, a given location on the cell surface is being distanced from the advancing pole at a slower rate, and hence accumulation starts at a closer distance from the tip if callose synthesis rates by individual synthases are otherwise unaltered. However, if this hypothesis holds true for both, callose of cellulose, the question arises why the two components are affected to a different degree. This difference could be explained with different basal rates of the synthesis activity. On the other hand, similar to cellulose, callose deposition might also be enhanced to compensate for the perceived increased turgor resulting from compression stressed caused by the centrifugal force.

The more puzzling phenomenon is the fact the callose layer becomes irregular and speckled at omnidirectional-*g*. Clearly, there is a disturbance in the deposition of this layer upon this supposedly gentler type of gravity-stress. Whether the irregular patches are a result of a reduced number of callose synthases being supplied, or their clustering within the plasma membrane, or yet another mechanism remains to be elucidated.

### Conclusion

To our knowledge, this is the first time fluorescence microscopy has been used to monitor live sub-cellular events in hyper-*g* conditions. Our novel approach allowed to shed light on the behaviour of non-statocyte plant cells in response to changes in gravitational acceleration, suggesting that cell wall assembly and intracellular trafficking are affected to compensate for the altered mechanical load imposed by omnidirectional-*g* and hyper-*g*. Although complementary experiments are required to understand the causality link between the different effects of altered gravitational acceleration, this approach proved the feasibility of live cell fluorescence imaging in hyper-*g* conditions.

## Supporting Information

Figure S1 **Spatial distribution profile of cellulose and callose.** Relative spatial distribution of cellulose (A) and callose (B) in *Camellia* pollen tubes grown at omnidirectional-*g* (orange), 1 *g* (blue), and hyper-*g* (2 *g*, 5 *g*, 7 *g*, 10 *g* and 14 *g*) represented by shades of red (cellulose) and green (callose) with darkest shade for highest *g*-value. Relative label intensities were quantified along the meridional tube surface measured on z-stack projections. All samples grown at hyper-*g* were significantly different from the samples grown at omnidirectional-*g* or at 1 *g,* but no difference was observed between the individual levels of hyper-*g*.
(TIF)

Figure S2 **Callose distribution under omnidirectional exposure to gravity.** Confocal laser scanning micrographs of a pollen tube grown in omnidirectional-*g* labelled with (1→3)-β-glucan against callose. The three micrographs were taken from the same region of the pollen tube at different focal depths. Arrows indicate where the optical section is positioned cortically to show the patchy distribution of callose in the cell wall. Bar = 10 µm.
(TIF)
