# Supplementary figures and images for: Cell Wall Assembly and Intracellular Trafficking in Plant Cells Are Directly Affected by Changes in the Magnitude of Gravitational Acceleration
Source: PLoS One. 2013 Mar 13;8(3):e58246. doi: 10.1371/journal.pone.0058246 (PMC3596410; doi:10.1371/journal.pone.0058246)

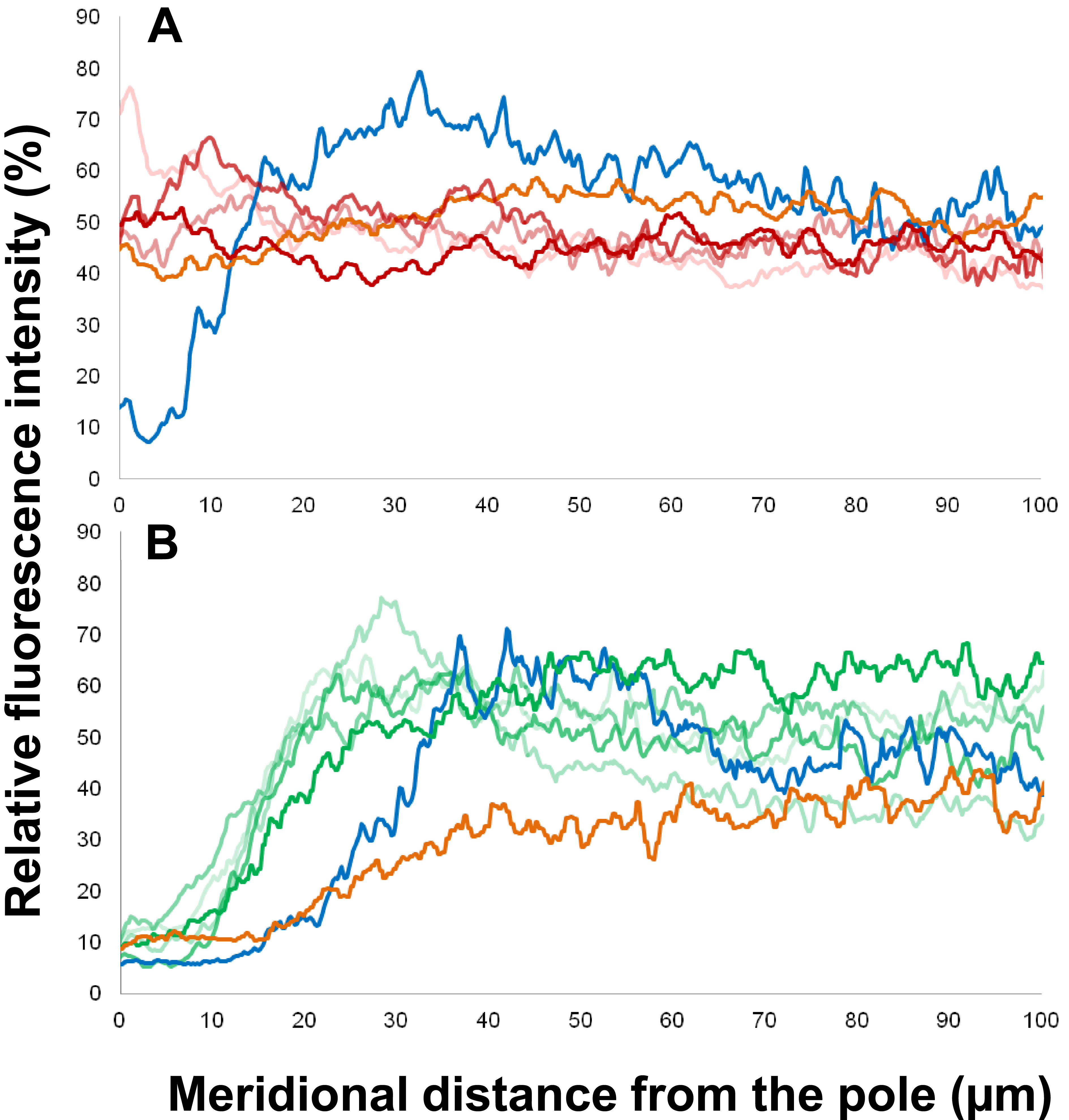

Supplement: Figure S1 — Spatial distribution profile of cellulose and callose. Relative spatial distribution of cellulose (A) and callose (B) in Camellia pollen tubes grown at omnidirectional-g (orange), 1 g (blue), and hyper-g (2 g, 5 g, 7 g, 10 g and 14 g) represented by shades of red (cellulose) and green (callose) with darkest shade for highest g-value. Relative label intensities were quantified along the meridional tube surface measured on z-stack projections. All samples grown at hyper-g were significantly different from the samples grown at omnidirectional-g or at 1 g, but no difference was observed between the individual levels of hyper-g. (TIF) [file pone.0058246.s001.tif]

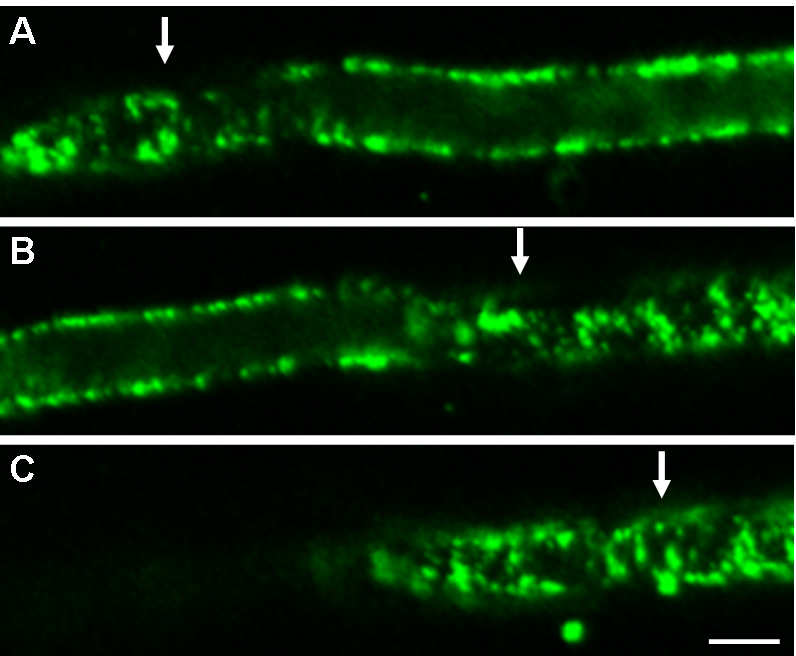

Supplement: Figure S2 — Callose distribution under omnidirectional exposure to gravity. Confocal laser scanning micrographs of a pollen tube grown in omnidirectional-g labelled with (1→3)-β-glucan against callose. The three micrographs were taken from the same region of the pollen tube at different focal depths. Arrows indicate where the optical section is positioned cortically to show the patchy distribution of callose in the cell wall. Bar = 10 µm. (TIF) [file pone.0058246.s002.tif]
